# Supramolecular Terbium‐Threonine Chiral Emitters with Tunable Circularly Polarized Luminescence as Anticounterfeiting‐Ink for Autonomous Data Encryption

**DOI:** 10.1002/advs.77336

**Published:** 2026-08-21

**Authors:** P. M. Ajay Sekhar, Nidhin Ravi, Kaloor S. Harikrishnan, Jim Bachmann, Nikos L. Doltsinis, Reji Varghese

**Affiliations:** ^1^ School of Chemistry Indian Institute of Science Education and Research (IISER) Thiruvananthapuram Thiruvananthapuram Kerala India; ^2^ Institut für Festkörpertheorie and Center for Multiscale Theory and Computation Universität Münster Münster Germany

**Keywords:** anticounterfeiting ink, circularly polarized luminescence (CPL), data encryption, dynamic systems, supramolecular materials

## Abstract

There has always been a growing demand for the development of advanced materials with dynamic properties for multi‐level data encryption and anticounterfeiting. Herein, we report a facile one‐pot, aqueous synthesis of a high nuclearity, luminescent, and circularly polarised luminescence (CPL)‐active terbium‐threonine supramolecular chiral emitters (**Tb‐Thr_D/L_‐G** NCs) from readily available precursors. An aqueous solution of **Tb‐Thr_D/L_‐G** NCs exhibits (i) 100% transparency, (ii) tunable luminescence and CPL, and (iii) pH feedback loop‐controlled “on‐off” of luminescence and CPL. By exploring these features, multi‐level data encryption with enhanced security is demonstrated. The remarkable features of our strategy include: (i) temporal control over encryption‐decryption, (ii) ability to expunge the stored information autonomously, and (iii) four distinct keys to decrypt the information, thereby offering enhanced security for the stored data. The potential of NCs as a covert anticounterfeiting ink for inkjet printing, and their excellent compatibility with commercially available polymer matrices such as polyvinyl alcohol, are also demonstrated. We believe that our rational strategy for the scalable synthesis of **Tb‐Thr_D/L_‐G** NCs with remarkable optical and chiroptical properties in aqueous media using readily available materials will pave the way for the facile synthesis of new lanthanide‐based chiral emitters with promising applications.

## Graphical abstract

A one‐pot, aqueous, and scalable method for synthesizing chiral lanthanide supramolecular emitters using terbium and threonine is reported. The aqueous solution of these chiral emitters displays the following features: (i) 100 % transparency, (ii) tunable luminescence and CPL, and (iii) a pH feedback loop that controls the “on‐off” states of luminescence and CPL. By leveraging these properties, we demonstrate high‐level data encryption that operates autonomously, as well as their potential use as biocompatible anticounterfeiting ink.

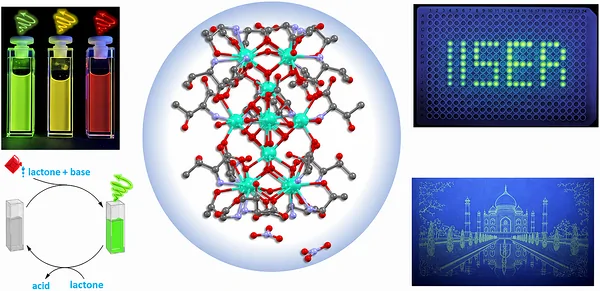

## 1 Introduction

The secure storage of information and anticounterfeiting of goods‐such as currency, medicine, and official documents‐are two critical issues that require urgent attention [1, 2]. In recent years, various materials have rapidly emerged to address these issues [3, 4]. The majority of these materials found in the literature and commercial markets primarily rely on a single fluorescence readout mechanism [5, 6]. This is typically achieved by incorporating fluorescent molecules or materials into polymers [7] or supramolecular matrices [8, 9, 10, 11, 12, 13], or by functionalizing with fluorescent tags [14]. By leveraging their tunability in emission wavelengths across multiple dimensions and exploring the stimuli‐responsive nature of fluorescent materials, researchers have achieved high‐level data encryption and anticounterfeiting measures [15, 16]. However, these approaches depend mainly on a single fluorescence readout as the key signal [17], which limits the level of security of the encrypted data [18]. Furthermore, issues like false positive fluorescence signals, multistep synthesis, and photostability hinder the effectiveness of fluorescence‐based data encryption. Therefore, to enhance the security of encrypted data and improve anticounterfeiting efforts, it is essential to develop dual or multi‐optical mode materials [19, 20, 21, 22].

Circularly polarized luminescence (CPL) is “chiral luminescence” and is due to the differential emission of left and right circularly polarized light [23]. This suggests that if a luminescent molecule is intrinsically chiral or located in a chiral environment, it can exhibit both luminescence and CPL, though not always [24, 25, 26, 27]. As a result, chiral luminophores not only provide signals based on luminescence but also deliver additional signals due to CPL. Most importantly, CPL signals are unique in many ways compared to luminescence, which include (i) nearly impossible to replicate, (ii) detection and imaging require specialized equipment, and (iii) high signal‐to‐noise ratio [28, 29]. Hence, chiral luminophores offer CPL‐based multidimensional protection for the encrypted data in addition to fluorescence [30]. For most organic molecules, the emission dissymmetry factor (g_lum_) is relatively low, ranging from 10^−5^ to 10^−3^, particularly in aqueous environments [31, 32, 33, 34]. In contrast, this factor is significantly higher for lanthanide complexes, ranging from 0.1 to > 1.0, due to magnetically allowed and electronically forbidden *f–f* electronic transitions [35, 36, 37]. Moreover, lanthanide‐based materials exhibit line‐like emission, significant Stokes shifts in their luminescence, long excited‐state lifetimes, high colour purity, and resistance to photobleaching [38, 39]. However, due to the low molar absorptivity of *f–f* transitions, their applications are limited without an appropriate ligand that can act as an antenna to sensitize the lanthanide [40]. Additionally, synthesizing crystalline lanthanide materials remains challenging due to their rapid hydrolysis in aqueous media [41]. The choice of a suitable chiral ligand can ensure sensitization, protection against excessive hydrolysis, and also chirality transfer to the lanthanide. Recent years have witnessed the rapid emergence of lanthanide‐based chiral emitters including perovskite films [42], peptide polymers [43], nanoparticles [44], MOFs [45], and liquid crystals [46] as powerful dual‐optical‐mode materials for multi‐level secure information storage and as highly effective anti‐counterfeiting agents [47, 48]. However, the lion's share of these materials were fabricated via a complex, tedious synthesis route, which hampers their real‐life applications. Hence, the development of a new class of chiral lanthanide emitters that exhibit (i) excellent luminescence, (ii) CPL signal with high g_lum_, and (iii) tuneable luminescence and CPL, in a less laborious and one‐pot synthesis manner, is indispensable for the advancement of their practical applications.

The security of stored data using chiral lanthanide emitters [49, 50, 51] can be significantly enhanced by customizing the system's programmability [52]. However, designing and synthesizing dynamic chiral lanthanide emitters is quite challenging due to the complexity of the process and the unreliability of the reversible processes. Despite these challenges, a few lanthanide‐based dynamic systems have been successfully developed that respond to external stimuli such as pH [53], solvent composition [54], light [55], and humidity [56]. These systems show promise for luminescence‐based temporal control of information storage and anti‐counterfeiting measures [5, 57]. It would be even more advantageous to create an autonomous dynamic system with multi‐optical readout capabilities for temporal control over information storage [58]. Such systems could offer a temporal readout of stored information and allow for the self‐erasure of information in an autonomous manner, making it inherently difficult to counterfeit. Recently, researchers have explored pH feedback loops to create self‐assembling systems that operate out‐of‐equilibrium, featuring programmable lifecycles and advanced self‐regulating materials with autonomous functions [59, 60, 61]. Hence, by integrating a pH feedback loop into a chiral lanthanide emitter, it may be possible to provide dynamic control over both optical and chiroptical readouts, which in turn permits the temporal control of stored information. However, developing a molecular or nano‐entity that encompasses these essential features–one‐pot synthesis using readily available inexpensive materials, scalability, biocompatibility, autonomous dynamics, covert operation, non‐destructive, multi‐optical readout, temporal control on encryption‐decryption, and self‐erasability‐remains a formidable challenge and has not yet been achieved.

**FIGURE 1:**
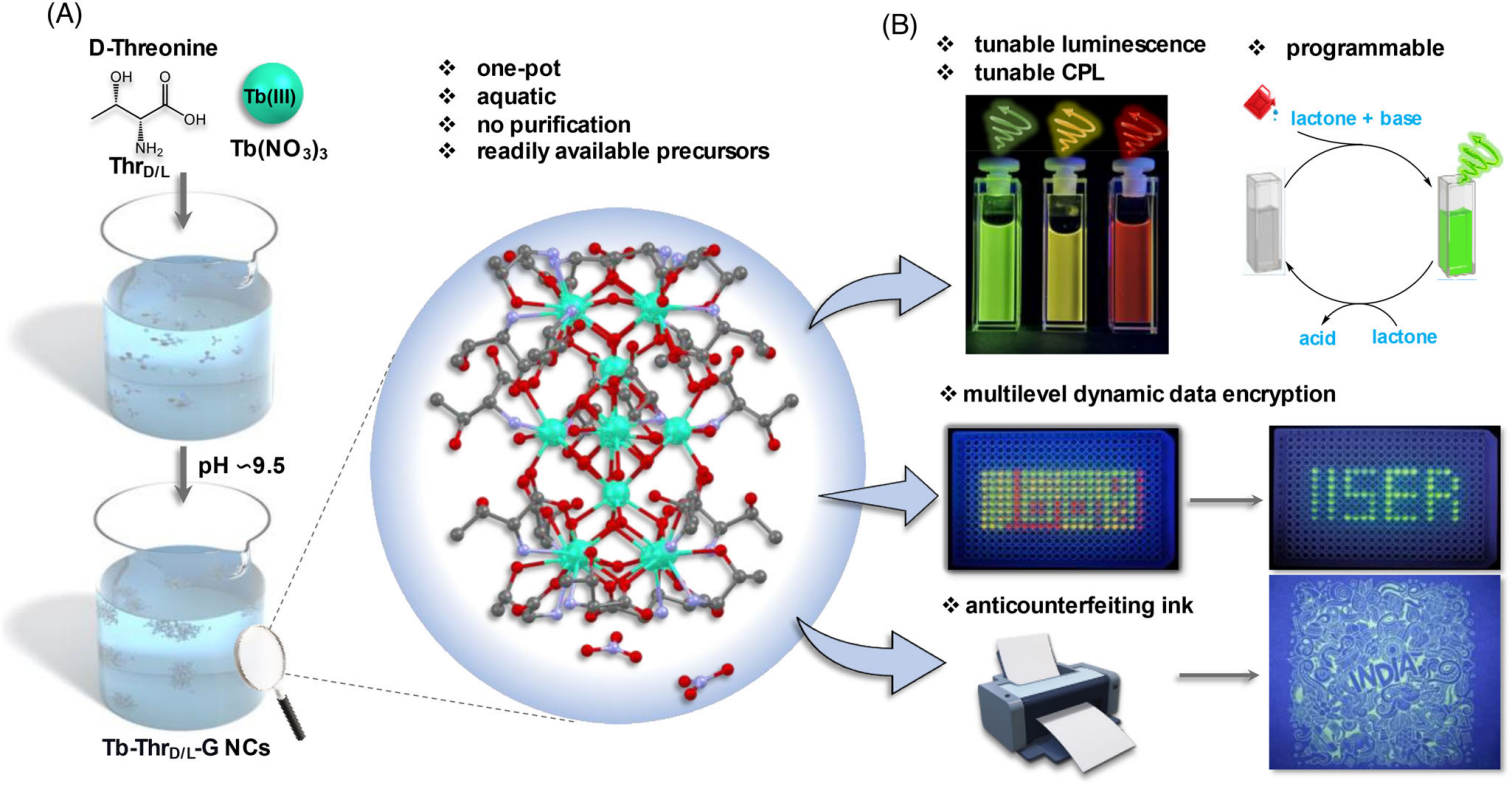
Synthesis of **Tb‐Thr_D/L_** NCs and their applications. (A) Scheme showing the one‐pot synthesis of **Tb‐Thr_D/L_** NCs in water. (B) Photograph of **Tb‐Thr_D/L_** NCs in water under UV illumination showing the tunability of luminescence and CPL by the controlled doping of **Eu(III)** (top panel, left). Schematic representation demonstrating the programmability of luminescence and CPL of **Tb‐Thr_D/L_** NCs with the addition of a mixture of lactone and base (top panel, right). Photograph displaying the luminescence patterns of **Tb‐Thr_D/L_‐G**, **Tb‐Thr_D_‐Y**, and **Tb‐Thr_D_‐R** NCs under UV irradiation, demonstrating the multi‐level data encryption (middle panel). Photograph taken under UV light of a print that uses **Tb‐Thr_D_‐G** as an anti‐counterfeiting ink (bottom panel).

Herein, we report a simple yet efficient one‐pot aqueous synthesis of terbium (**Tb**)‐threonine (**Thr_D/L_**) high nuclearity nanocrystals (**Tb‐Thr_D/L_**) using inexpensive and readily available starting materials, terbium nitrate [Tb(NO_3_)_3_] and **Thr_D/L_**, at a basic pH of ∼9.5. The NCs are crystallized in a chiral space group, and their structure has been fully determined using single‐crystal X‐ray diffraction (SCXRD). The **Tb‐Thr_D/L_** nanocrystals (NCs) in water are nearly 100% transparent and do not absorb visible light, making them undetectable in daylight. The NCs display the characteristic intense luminescence of **Tb(III)**. Due to the chiral nature of the NCs, they also exhibit commendable CPL activity, boasting a high |g_lum_| of 0.03, which is impressive for **Tb(III)**‐based NCs in aqueous media [33]. Another notable feature of the NCs is the tunability of their luminescence and CPL properties through controlled **Eu(III)** doping. This allows for the synthesis of yellow (**Tb‐Thr_D/L_‐Y**) and red (**Tb‐Thr_D/L_‐R**)‐luminescent and CPL‐active NCs by doping **Tb‐Thr_D/L_‐G** NCs with 5% and 10% **Eu(III)**, respectively. Subsequently, with the integration of a pH feedback loop into the chiral **Tb‐Thr_D/L_‐G** emitters, we demonstrate the temporal control over the “on” and “off” states of the optical and chiroptical properties in an autonomous fashion. This combination of exceptional optical and chiroptical properties, along with pH feedback‐driven programmability, has enabled the achievement of advanced multilevel data encryption and anti‐counterfeiting measures. An aqueous solution of NCs is completely transparent, making any encrypted information undetectable in daylight. By adding a mixture of a lactone (ε‐caprolactone or β‐butyrolactone) and a base (triethylamine or sodium hydroxide) as a chemical additive, the optical and chiroptical properties of NCs are activated in a wavelength‐tuneable fashion under UV light. This process introduces additional layers of security for the stored information. Most importantly, decrypting the CPL information requires sophisticated instrumentation, making the encryption highly secure. Moreover, pH‐feedback‐based programmability of the system provides a predefined lifetime for the luminescence and CPL signals by optimizing the concentrations of the chemical additives, enabling temporal control over the encryption. Eventually, the self‐destructive feature erases all stored data, returning to the original undetectable state autonomously. Our method offers high‐level security for stored data, which can only be accessed using four distinct keys: UV light, chemical additive, time, and CPL. Furthermore, the material can be used as an anti‐counterfeiting ink with commercial inkjet printers. Highly complex patterns and symbols can be printed using **Tb‐Thr_D/L_‐G** NCs in water as the ink, remaining fully invisible to the naked eye. However, these printed details become vividly visible under UV light irradiation. Our findings suggest that NCs possess essential characteristics for effective data encryption and serve as an anti‐counterfeiting agent within a single nanosystem, a feature that is difficult to achieve (Figure 1).

## 2 Results

### 2.1 Synthesis and Characterization of Tb‐Thr_D/L_‐G NCs

The synthesis of lanthanide complexes in water is usually performed at acidic or physiological pH to prevent a high hydrolysis rate at the metal centre [62]. However, these conditions typically result in non‐luminescent complexes due to water coordination [63, 64]. On the other hand, synthesizing complexes at basic pH often leads to the formation of non‐crystalline, non‐stoichiometric, and non‐luminescent complexes due to the high hydrolysis rate at the metal centre [65, 66]. These undesirable properties significantly limit their practical applications. Therefore, it is extremely challenging to synthesize crystalline lanthanide complexes in water at high basic pH while preserving the luminescent properties of the metal ions. Biomolecules [67, 68], particularly amino acids [69], have been utilized as ligands for the synthesis of lanthanide complexes due to their excellent biocompatibility and multiple binding sites. We hypothesize that at high basic pH (>9), both the amine and carboxylate groups of amino acids can simultaneously coordinate with the metal centre. This coordination could potentially exclude water and hydroxyl ions from the first coordination sphere, thereby preventing the extensive hydrolysis at the metal centre. This potentially preserves the optical properties of the lanthanide ions intact. In order to optimize a suitable pH for the synthesis, pH‐dependent synthesis was performed, and the formation of a strongly luminescent material was observed only at pH ∼9.5. Hence, the lanthanide‐amino acid complex was synthesized at pH ∼9.5. For our study, we selected **Tb(III)** as the lanthanide ion because of its promising luminescence properties, and **Thr** as the chiral ligand due to its multiple binding sites (alcohol, amine, and carboxylate groups). Our synthesis involved simple mixing of **Thr_D/L_** (92 mM) and Tb(NO_3_)_3_ (46 mM) in water at 25°C. The pH of the solution was maintained at ∼9.5 by adding triethylamine (10 µL) (Figure 1A) or sodium hydroxide. This reaction resulted in the spontaneous formation of green‐emissive **Tb‐Thr_D/L_** NCs (**Tb‐Thr_D/L_‐G** NCs) in water, with no further purification required. It is important to note that the formation of green‐emissive NCs was observed only at a basic pH of ∼9.5, and the luminescent NCs gradually disappeared as the solution pH decreased or increased. The unique characteristics of our synthesis include: (i) aquatic, (ii) one‐pot process, (iii) readily available and inexpensive precursors, and (iv) no purification step required.

**FIGURE 2:**
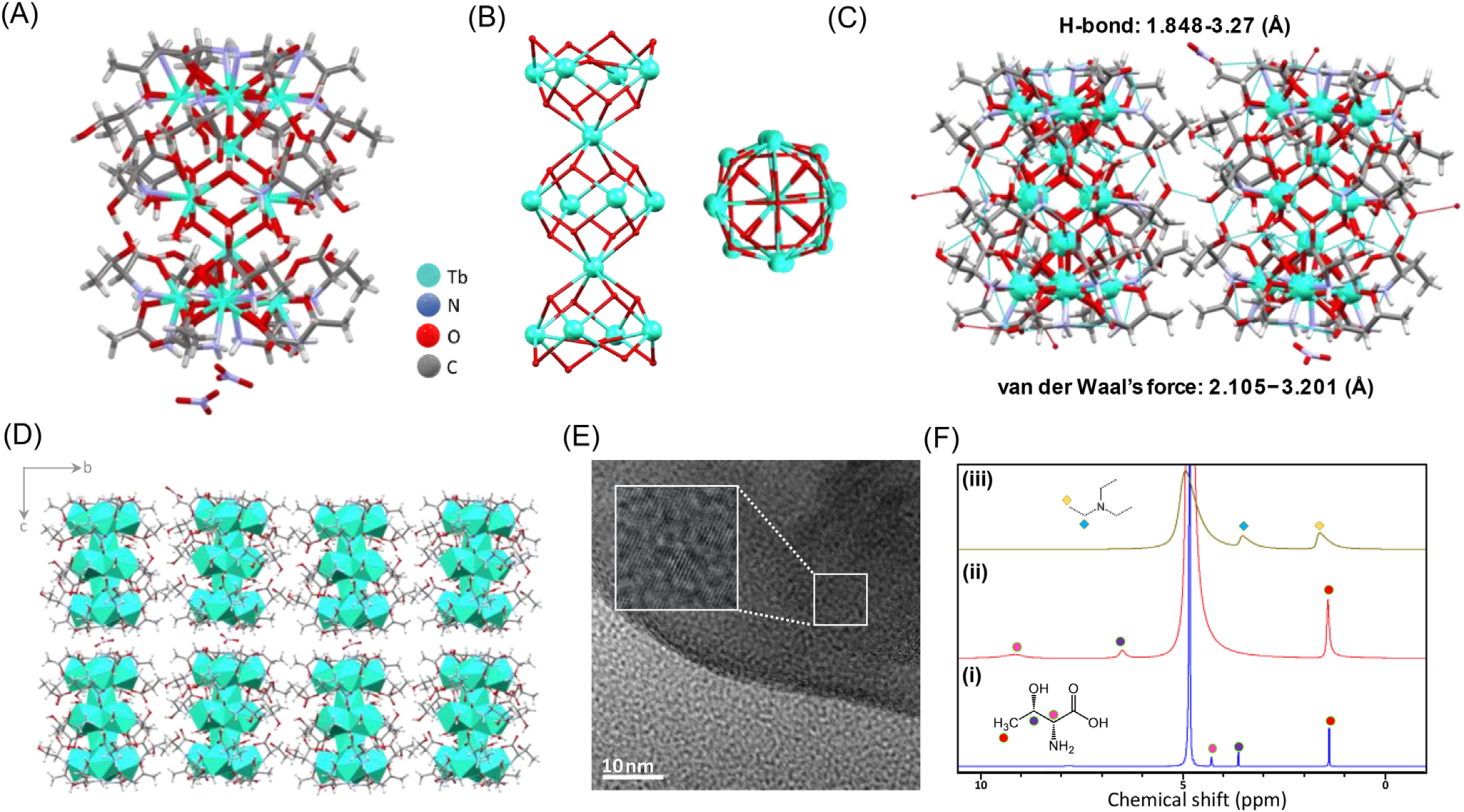
Characterization of **Tb‐Thr_D_‐G** NCs. (A, B) X‐Ray crystal structure of **Tb‐Thr_D_‐G** NC. Tb, N, O, and C are represented by cyan, light violet, red, and grey, respectively. (C) Showing the various hydrogen bonding and van der Waals interactions present in the crystal. (D) Self‐assembly of **Tb‐Thr_D_‐G** unit in the crystal. Solvent molecules were squeezed for clarity. (E) HR‐TEM image of **Tb‐Thr_D_‐G** NCs. (F) Comparison of ^1^H‐NMR spectra (D_2_O, 500 MHz, 25°C) of (i) **Thr_D_** at pH ∼4.5, (ii) Tb(NO_3_)_3_ and **Thr_D_** at pH ∼4.5, and (iii) Tb(NO_3_)_3_ and **Thr_D_** at pH ∼9.5 with the addition of triethylamine.

We could grow diffraction‐quality single crystals of **Tb‐Thr_D_‐G** by allowing acetone vapour to diffuse into the aqueous solution of **Tb‐Thr_D_‐G** over a period of four weeks. Single‐crystal analyses of ***Tb‐Thr_D_*‐*G*** NCs revealed that it crystallises in the orthorhombic crystal system with a chiral space group P2**_1_** 2**_1_** 2 and a basal building unit of [Tb_14_(C_4_H_9_NO_3_)_20_(OH)_16_(O)_2_(H_2_O)_8_]^2+^. Each ***Tb‐Thr_D_‐G*** NC consists of fourteen **Tb(III)**, twenty **Thr_D_**, eight water molecules, sixteen hydroxyl groups, two oxygen atoms, and two nitrate anions (Figure 2A). The fourteen **Tb(III) ions** are arranged in a tetradecanuclear cluster, comprising three layers of tetrameric square **Tb(III)** ions sandwiched between two layers of single **Tb(III)** ions. The top and bottom tetrameric **Tb(III)** layers are amalgamated via an η^4^‐oxo ligand (Figure 2B). The metal core is interconnected by triply bridging hydroxo ligands, and **Thr_D_** protects the peripheral region of the NCs from excessive hydrolysis through multidentate chelate coordination of its amine, alcohol, and carboxylate groups. There are three different coordination environments for **Tb(III)** in the NCs, each with a coordination number of eight. The **Tb(III)**−**Tb(III)** distance between the neighboring ions in the tetrameric square ranges from 3.69 to 3.89 Å, while the distance between the tetrameric square and the middle single **Tb(III)** layer ranges from 3.76 to 3.87 Å. The major non‐covalent interactions driving the growth of the NCs in all possible directions are H‐bonding and van der Waals interactions, with distances of 1.848–3.27 Å and 2.105–3.201 Å, respectively (Figure 2C,D). High‐resolution transmission electron microscopic (HR‐TEM) images also showed high crystallinity for **Tb‐Thr_D/L_‐G** NCs (Figure 2E). In accordance with the crystal structure of **Tb‐Thr_D_‐G** NCs, the ^1^H NMR spectrum of **Tb‐Thr_D_‐G** NCs (40 mg/mL Tb(NO_3_)_3_ and 20 mg/mL **Thr_D_**, D_2_O, pH ∼9.5) showed significant downfield shifts for the hydrogens of **Thr_D_**, confirming its coordination to the paramagnetic **Tb(III)** (Figure 2F). This observation also validates the structural integrity of **Tb‐Thr_D_‐G** NCs in the solution state.

### 2.2 Optical, Chiroptical and Computational Studies of Tb‐Thr_D/L_‐G NCs

**FIGURE 3:**
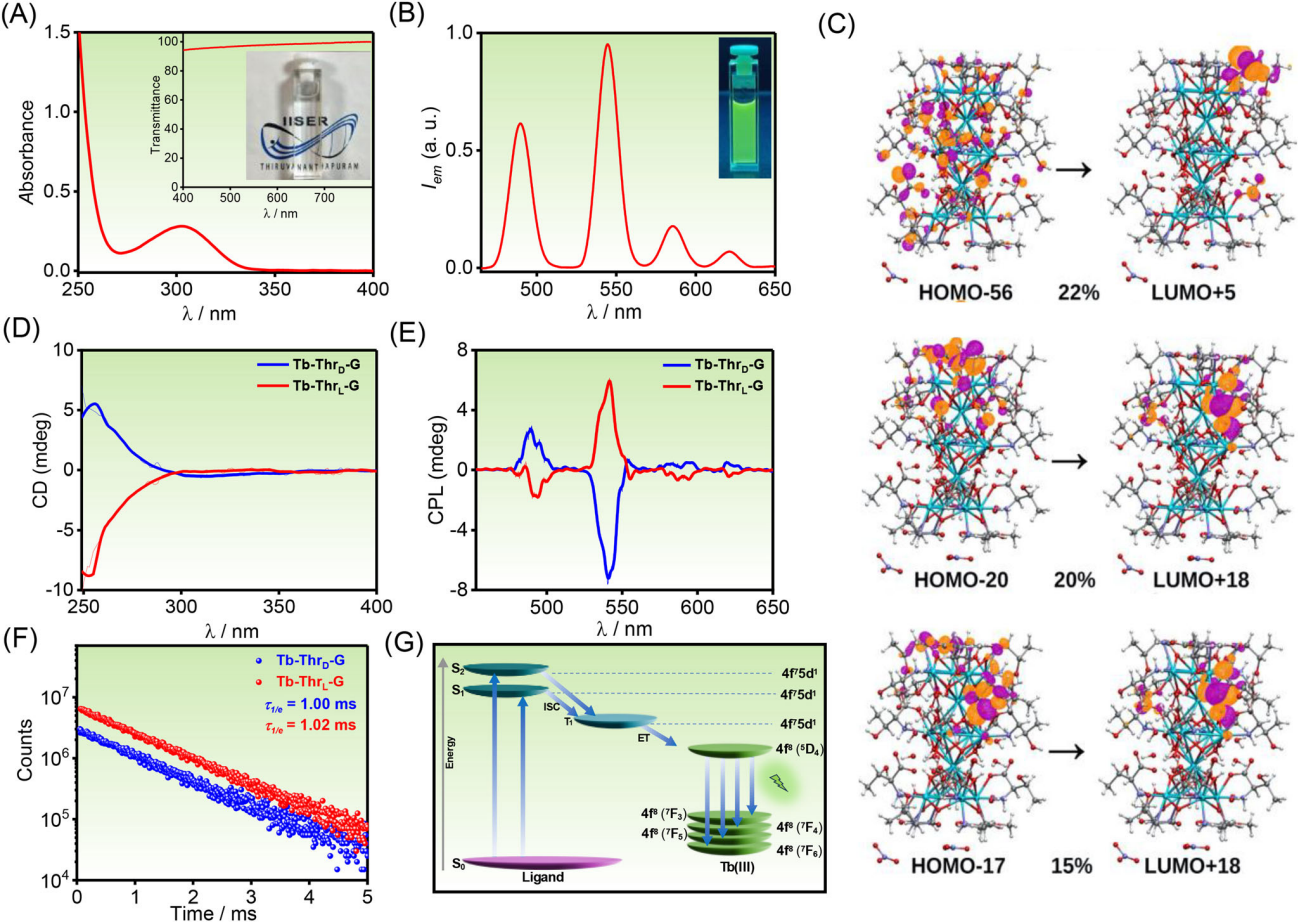
Optical, chiroptical, and computational studies of **Tb‐Thr_D/L_‐G NCs**. (A) Absorption spectrum of **Tb‐Thr_D_‐G** NCs in water (20 mg/mL: Tb(NO_3_)_3_, **Thr_D/L_**: 10 mg/mL, pH ∼9.5), and the inset shows the corresponding transmittance spectrum. An image of a cuvette containing **Tb‐Thr_D/L_‐G** NCs in water to demonstrate the 100% transparent nature of the NCs solution is shown. (B) Emission spectrum of **Tb‐Thr_D/L_‐G** NCs in water, and the inset shows the photograph of the corresponding solution under UV irradiation. (C) Dominant orbitals contributing to the high‐intensity cluster transition with a wavelength around 250 nm. (D) The CD spectra of **Tb‐Thr_D_‐G** (blue) and of **Tb‐Thr_L_‐G** (red) NCs in water (40 mg/mL: Tb(NO_3_)_3_, **Thr_D/L_**: 20 mg/mL, pH ∼9.5), and (E) the corresponding CPL spectra (λ_exc_ = 255 nm). **(F)** Time‐resolved photoluminescence spectra of **Tb‐Thr_D/L_‐G** NCs in water collected at 545 nm (λ_exc_ = 255 nm). (G) A plausible Jablonski diagram depicting the possible electronic transitions for the NCs.

All the optical and chiroptical studies of **Tb‐Thr_D/L_‐G** NCs were performed in water at pH ∼9.5. The absorption spectrum of **Tb‐Thr_D/L_‐G** NCs (20 mg/mL: Tb(NO_3_)_3_, 10 mg/mL: **Thr_D/L_**) displayed two bands centred at 300 and 255 nm (Figure 3A). These bands at 300 and 255 nm are attributed to amine‐to‐metal and carboxylate‐to‐metal charge transfer, respectively [70]. Notably, no absorption was observed in the entire visible region (400–800 nm), and hence **Tb‐Thr_D/L_‐G** NCs in water exhibited nearly 100% transparency (Figure 3A inset).

This observation was further confirmed by placing a solution of the NCs in front of the “IISER logo,” which remained clearly visible through the solution, just as it did outside of it (Figure 3A inset). As a result, the NCs are virtually undetectable to the naked eye under daylight, making them highly promising for applications in high‐level data encryption and anticounterfeiting. The luminescence spectrum (λ_exc_ = 255 nm) of **Tb‐Thr_D/L_‐G** NCs displayed the characteristic luminescence of **Tb(III)** with peaks at 490, 545, 583, and 620 nm, corresponding to the electronic transition from ^5^D_4_ to ^7^F_J_ (J = 6−3) (Figure 3B), with CIE coordinates of (0.29, 0.56) (Figure S2). The luminescence quantum yields of **Tb‐Thr_D_‐G** and **Tb‐Thr_L_‐G** NCs in water are 21% and 22%, respectively, and the corresponding lifetime values are 1.03 and 1.05 ms, respectively (Figure 3F). To validate the optical properties, we have performed time‐dependent density functional theory (TDDFT) calculations (general characterization for details). The calculated absorption spectrum (Figure S4) agrees well with the experimental data, featuring two bands centered at 250 and 300 nm. The most dominant orbital contributions to these peaks are given in Tables S3 and S4 and visualized in Figure 3C and Figure S6. Based on the orbital structure, it is evident that the 250 nm excitation originates from the ligands and culminates at the d‐orbitals of **Tb(III)**. This indicates the potential of the ligand to function as an antenna (Figure 3C) [40]. From these results, a plausible Jablonski‐type diagram is proposed to illustrate the processes involved in absorption and subsequent electronic relaxation (Figure 3G). The observed emission lines are derived from the f‐orbitals of **Tb(III)**, specifically transitions between ^5^D_4_ and ^7^F_3−6_ states. These conclusions align well with the reported **Tb(III)** emission [71]. Furthermore, the orbital analyses revealed that the absorption and, consequently, the luminescence involve multiple **Tb(III)** atoms.

**FIGURE 4:**
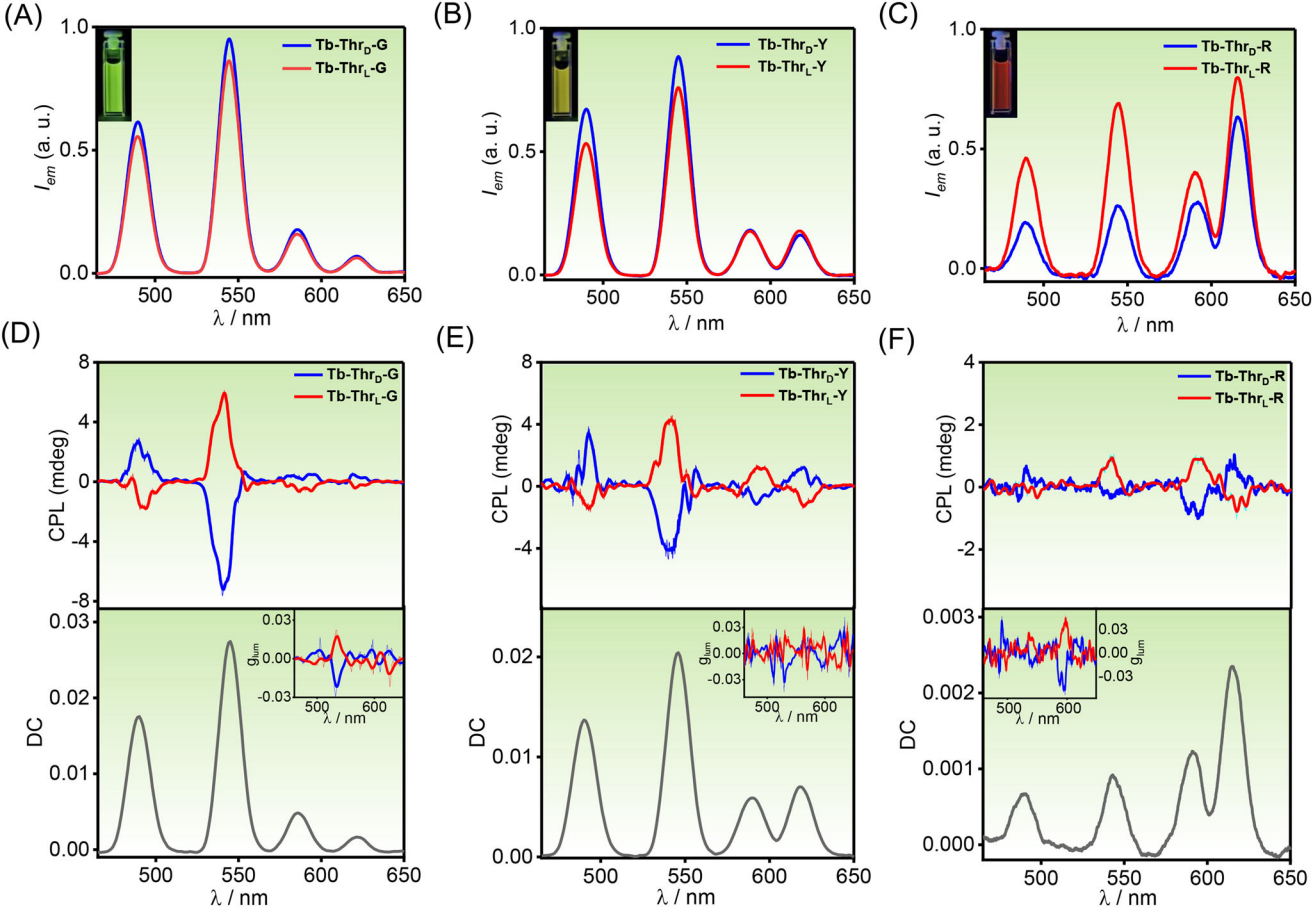
Tuning of luminesce and CPL of Tb‐Thr_D/L_‐G NCs by Eu(III) doping. Emission spectra (λ_ex_ = 255 nm, pH ∼9.5) of (A) Tb‐Thr_D/L_‐G, (B) Tb‐Thr_D/L_‐Y, and (C) Tb‐Thr_D/L_‐R NCs. The insets of the emission spectra show the photographs of the corresponding solutions under UV irradiation. The CPL and corresponding DC spectra (λ_ex_ = 255 nm, pH ∼9.5) of (D) Tb‐Thr_D_‐G (blue) and Tb‐Thr_L_‐G (red) NCs, (E) Tb‐Thr_D_‐Y (blue) and Tb‐Thr_L_‐Y (red), and (F) Tb‐Thr_D_‐R (blue) and Tb‐Thr_L_‐R (red) NCs. The insets of DC spectra show the g_lum_ values. [Tb‐Thr_D/L_‐G] = 40 mg/mL: Tb(NO_3_)_3_, Thr_D/L_: 20 mg/mL; [Tb‐Thr_D/L_‐Y] = 37.9 mg/mL: Tb(NO_3_)_3_, Eu(NO_3_)_3_: 2.1 mg/mL, Thr_D/L_: 20 mg/mL and; [Tb‐Thr_D/L_‐R] = 35.9 mg/mL: Tb(NO_3_)_3_, Eu(NO_3_)_3_: 4.1 mg/mL, Thr_D/L_: 20 mg/mL.

The circular dichroism (CD) spectrum of **Tb‐Thr_D_‐G** NCs (40 mg/mL: Tb(NO_3_)_3_, **Thr_D_**: 20 mg/mL) revealed a positive Cotton Effect centred at 255 nm. As expected, the corresponding **Tb‐Thr_L_‐G** NCs displayed a mirror image CD spectrum, featuring a negative Cotton Effect with a maximum centred at 255 nm (Figure 3D), and hence an excitation wavelength of 255 nm was used for the circularly polarized luminescence (CPL) studies. The CPL spectrum of **Tb‐Thr_D_‐G** NCs showed signals corresponding to all the luminescent bands at 490 nm (^5^D_4_ → ^7^F_6_), 545 nm (^5^D_4_ → ^7^F_5_), 583 nm (^5^D_4_ → ^7^F_4_), and 620 nm (^5^D_4_ → ^7^F_3_). Additionally, mirror image CPL signals were observed for all these bands in the corresponding CPL spectrum of **Tb‐Thr_L_‐G** NCs (Figure 3E). Interestingly, high CPL dissymmetry factors (|g_lum_|) of 0.006/0.005 (^5^D_4_ → ^7^F_6_), 0.030/0.026 (^5^D_4_ → ^7^F_5_), 0.021/0.019 (^5^D_4_ → ^7^F_4_) and 0.015/0.018 (^5^D_4_ → ^7^F_3_) were observed for **Tb‐Thr_D/L_‐G** NCs. These high g_lum_ values are particularly commendable considering the NCs were dispersed in the aqueous medium [34, 72]. Another remarkable feature of the NCs is the tunability of their optical and chiroptical properties by the controlled doping with **Eu(III)**. Doping of **Tb‐Thr_D/L_‐G** NCs (Figure 4A) in water with 5% and 10% of **Eu(III)** [Eu(NO_3_)_3_] resulted in the formation of yellow (**Tb‐Thr_D/L_‐Y**) (Figure 4B) and red (**Tb‐Thr_D/L_‐R**) (Figure 4C) emissive NCs, respectively. In addition, mirror image CPL signals were observed for all the luminescent bands of **Tb‐Thr_D/L_‐Y** and **Tb‐Thr_D/L_‐R** NCs (Figure 4D–F and Figure S7). The corresponding g_lum_ values are provided in the inset of Figure 4D–F. To get better insight into the structure of the nanocrystals after the Eu(III) doping, we have carried out powder X‐ray diffraction (PXRD) analyses of the undoped **Tb‐Thr_D_‐G** NCs as well as the Eu(III)‐doped samples, **Tb‐Thr_D_‐Y** and **Tb‐Thr_D_‐R** NCs (Figure S8). For these measurements, the samples were prepared by lyophilizing their respective aqueous solutions. As expected, the PXRD pattern of the lyophilized **Tb‐Thr_D_‐G** sample closely matches the simulated PXRD pattern generated from its single‐crystal X‐ray structure, suggesting that the crystal structure of **Tb‐Thr_D_‐G** is most likely retained in the solution state [35]. More importantly, the PXRD patterns of the Eu(III)‐doped nanocrystals, **Tb‐Thr_D_‐Y** and **Tb‐Thr_D_‐R**, were also nearly identical to that of **Tb‐Thr_D_‐G**. The close resemblance among the diffraction peaks of **Tb‐Thr_D_‐G**, **Tb‐Thr_D_‐Y**, and **Tb‐Thr_D_‐R** indicates that Eu(III) doping does not disrupt the crystalline framework of **Tb‐Thr_D_‐G** NCs and that the overall crystal architecture is preserved even after doping. To further elucidate the fate of Eu(III) following the doping with **Tb‐Thr_D_‐G** NCs, we performed time‐resolved luminescence lifetime measurements on **Tb‐Thr_D_‐G** and the Eu(III)‐doped **Tb‐Thr_D_‐Y** and **Tb‐Thr_D_‐R** NCs (Figure S9). The luminescence lifetime of **Tb‐Thr_D_‐G** was determined to be ∼1 ms. Notably, the Eu(III)‐doped **Tb‐Thr_D_‐Y** and **Tb‐Thr_D_‐R** NCs also exhibited a lifetime of ∼1 ms, with no discernible change compared to the undoped sample. The absence of any significant change in the Tb(III) luminescence lifetime indicates that there is negligible electronic interaction between Tb(III) and Eu(III) [39]. This observation supports our hypothesis that Eu(III) is not incorporated into the crystal lattice of **Tb‐Thr_D_‐G** during the doping. Taken together, the PXRD and luminescence lifetime results suggest that Eu(III) doping does not result in the incorporation of Eu(III) into the crystalline framework of **Tb‐Thr_D_‐G**. Instead, Eu(III) is likely to reside in the close vicinity of the nanocrystal surface or within the surrounding coordination environment without perturbing the crystal structure. We must, however, acknowledge that the currently available experimental data do not allow us to unambiguously determine the precise location or coordination environment of the Eu(III) ions relative to **Tb‐Thr_D_‐G** NCs.

Furthermore, in order to validate the structural robustness of the nanocrystals in the solution phase, we have performed optical (Figure S10) and chiroptical (Figure S11) measurements in the solid state and compared them with those in the solution phase. Accordingly, we recorded the solid‐state UV–vis absorption, luminescence, CD, and CPL spectra of **Tb‐Thr_D_‐G/Y/R** NCs. Encouragingly, the absorption, luminescence, CD, and CPL spectra of the solid‐state sample are nearly identical to those obtained in aqueous solution. These results suggest that the crystalline phase of the nanocomplex is preserved in the solution state. We have also performed xTB simulations of **Tb‐Thr_D_‐G** NC in aqueous solution. Notably, no significant structural deformation was observed throughout the 37.4 ps simulation, demonstrating the structural stability of the nanocomplex under aqueous solution conditions (Figure S12).

### 2.3 Programmable Optical and Chiroptical Properties of Tb‐Thr_D/L_‐G NCs

**FIGURE 5:**
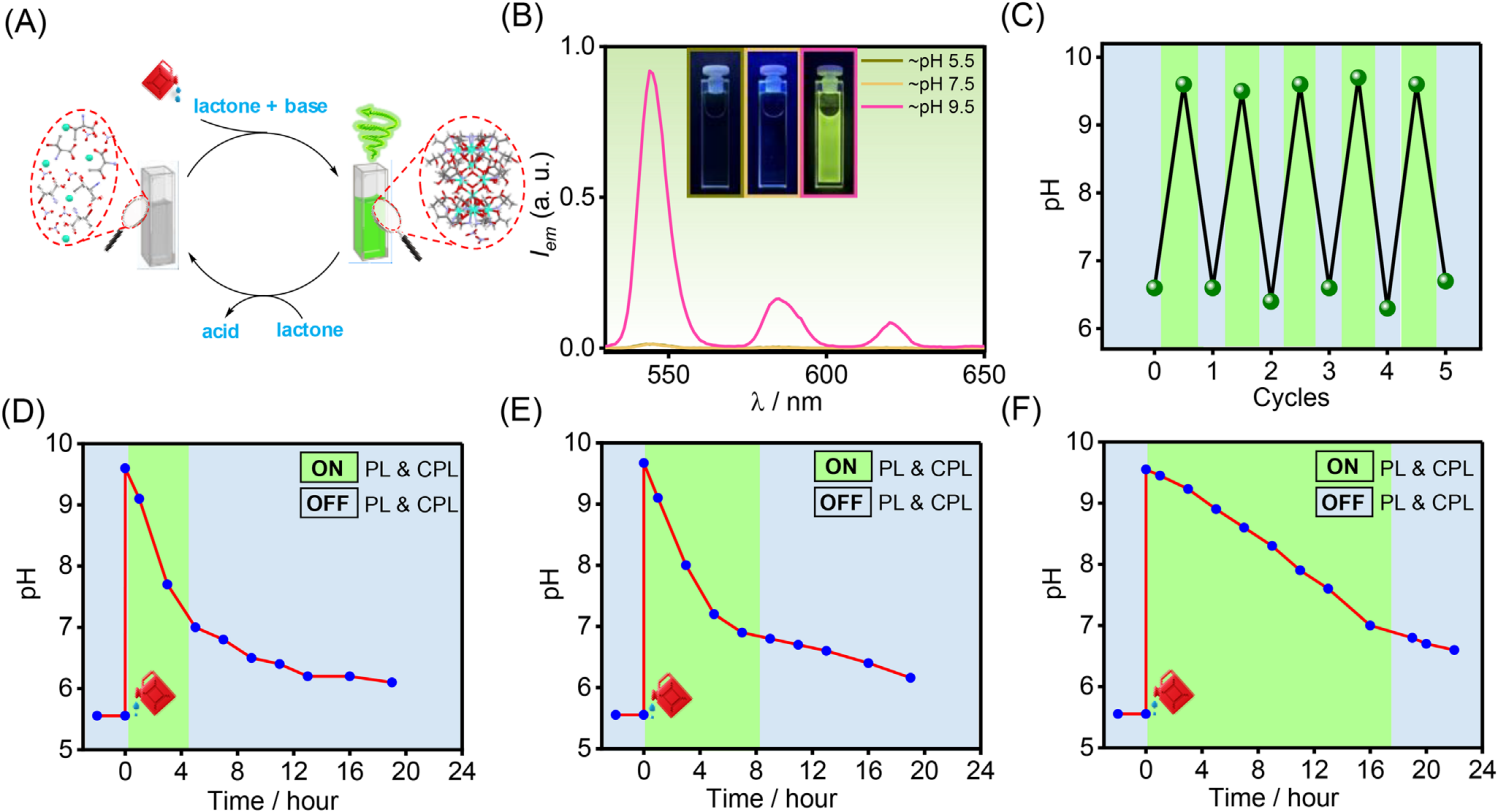
Programmable optical and chiroptical properties of **Tb‐Thr_D/L_‐G NCs**. (A) Schematic illustration depicting the temporal control over the turn “on‐off” of luminescence and CPL with the addition of a mixture of ε‐caprolactone and triethylamine as chemical additives. (B) Emission spectra of **Tb‐Thr_D_‐G** NCs in water (λ_exc_ = 255 nm) at different pH (∼5.5, ∼7.5, and ∼9.5), and the inset shows the photograph of the corresponding solutions under UV irradiation. (C) Luminescence and CPL “on‐off” cycles monitored at 545 nm with the addition of a mixture of ε‐caprolactone and triethylamine to **Tb‐Thr_D_‐G** NCs in water. Time‐dependent luminescence and CPL changes were monitored at 545 nm with the addition of varying concentrations of caprolactone (D) 160 µL, (E) 80 µL, (F) 40 µL, keeping the triethylamine volume the same (20 µL) to **Tb‐Thr_D_‐G** NCs in water.

The control over the spatiotemporal properties of natural systems has garnered significant attention due to their extraordinary outcomes, including autonomous functions [73], reversibility [74], self‐healing [75], and self‐erasing [76] abilities. Emulating these natural systems to create materials that exhibit self‐erasing behavior is beneficial in the field of data encryption, as it allows encrypted data to have predefined lifespans. Besides, such materials make counterfeiting much more difficult. However, developing a reliable and reproducible process that guarantees autonomous self‐erasing capabilities remains a challenge [77]. Here, we explore a pH feedback loop approach to achieve an autonomous “on‐off” cycle for the optical and chiroptical properties of **Tb‐Thr_D/L_‐G** NCs. The **Tb‐Thr_D/L_‐G** NCs synthesis was found to be highly sensitive to the pH of the solution, and the formation of the luminescent NCs was found mainly at pH ∼9.5. This is due to the effective coordination of the amine and carboxylate groups of **Thr** to **Tb(III)** at this pH, as evident from the crystal structure. A decrease in pH (pH 5 and 7) results in the protonation of the amine group (pKa ∼9.0), which in turn prevents the coordination of the amine to the metal centre. This leads to dissociation of the NCs into their corresponding non‐luminescent precursors. In support of this, crystallization at pH below 9 (specifically pH 5 and 7) yielded only the crystals of threonine (Figure S13), and no **Tb‐Thr_D_‐G** NCs were formed. Formation of the NCs “turns on” all their optical and chiroptical properties, whereas their dissociation at neutral or acidic pH “turns off” all optical and chiroptical properties. This suggests that by regulating the pH of the solution, the formation and dissociation of the NCs can be reversibly programmed. We have used a mixture of a lactone (eg: ε‐caprolactone) and a base (eg: triethylamine) as chemical additives to achieve the programmability, which regulates the pH of the solution as an internal feedback system in a predictable and autonomous manner (Figure 5A). For this, a mixture of **Thr_D/L_** (5.47 mg, 2 eq.) and Tb(NO_3_)_3_ (10 mg, 1 eq.) was prepared in deionized water (1 mL). A mixture of triethylamine (10 µL) and ε‐caprolactone (60 µL) was separately prepared and added to the **Thr_D/L_** and Tb(NO_3_)_3_ mixture to regulate the solution pH over time. Initially, the sudden rise in pH of the solution (pH ∼9.5) caused the spontaneous formation of **Tb‐Thr_D/L_‐G** NCs, which “turn on” their optical and chiroptical properties. At the same time, caprolactone underwent slow hydrolysis in water, producing hydroxycarboxylic acid (6‐hydroxycaproic acid). This reaction gradually decreased the pH of the solution, leading to the dissociation of the NCs over time and “turning off” their optical and chiroptical properties (Figure 5B and Figure S14). This “on‐off” cycle can be repeated for several cycles by adding a new batch of the chemical additives (Figure 5C). More importantly, the programmability of the system can be adjusted by varying the concentration of the chemical additive. For instance, a higher concentration of ε‐caprolactone resulted in a shorter emissive state, while a lower concentration produced a longer emissive state. By precisely controlling the concentration of ε‐caprolactone, we were able to fine‐tune the lifetime of the emissive state, extending it from minutes to hours. For example, concentrations of ε‐caprolactone of 160 (Figure 5D), 80 (Figure 5E), and 40 (Figure 5F) µL resulted in emissive state lifetimes of 4, 8, and 17 h, respectively. Additionally, programmability can be tuned by the selection of lactones. δ‐Gluconolactone and β‐butyrolactone are known to hydrolyze faster than ε‐caprolactone to the corresponding carboxylic acid [54]. By using the same concentration (400 mM) of δ‐gluconolactone, β‐butyrolactone, and ε‐caprolactone, we successfully tuned the lifetimes of the emissive states to 10, 100, and 150 min, respectively.

### 2.4 Multilevel Data Encryption Application

**FIGURE 6:**
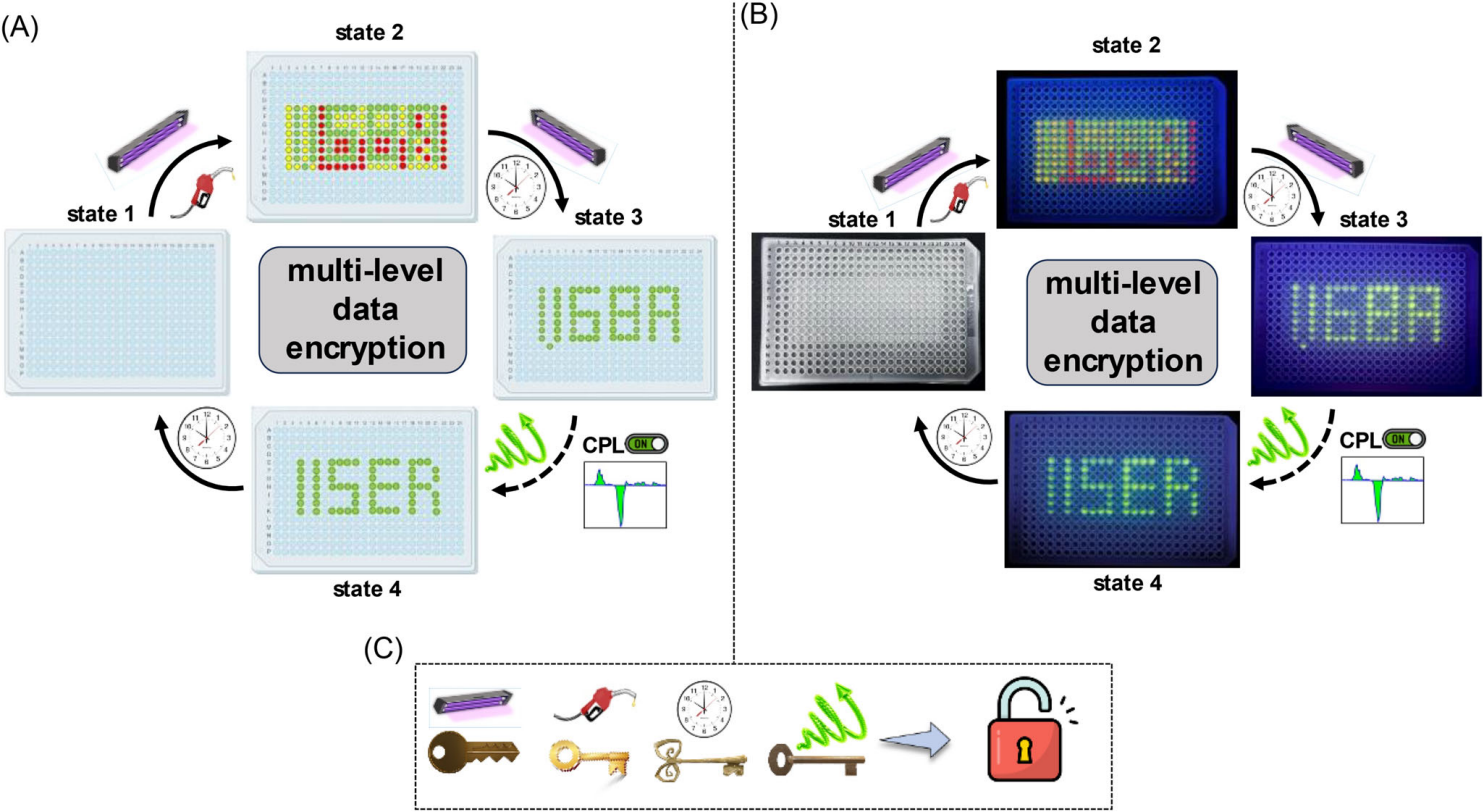
Multilevel data encryption using the NCs. (A) Schematic illustration of multilevel and dynamic information encryption using Tb‐Thr_D/L_‐G, Tb‐Thr_D_‐Y, and Tb‐Thr_D_‐R NCs and the decryption of the information using four different keys, including UV, chemical additives, time, and CPL, and (B) the corresponding proof‐of‐concept experiment. (C) Schematic representation of the four different keys used to decrypt the information.

After establishing the programmability of the optical and chiroptical properties of **Tb‐Thr_D/L_‐G** NCs, we demonstrated their potential for multi‐level data encryption, and a pattern of “IISER” was used for this purpose (Figure 6A). Mixtures of (i) Tb(NO_3_)_3_ (180 mM) and **Thr_D_** (360 mM) (green emission, D‐isomer), (ii) Tb(NO_3_)_3_ (180 mM) and **Thr_L_** (360 mM) (green emission, L‐isomer), (iii) Tb(NO_3_)_3_ (175 mM), **Thr_D_** (360 mM) and Eu(NO_3_)_3_ (9 mM) (yellow emission, D‐isomer), and (iv) Tb(NO_3_)_3_ (165 mM), **Thr_D_** (360 mM) and Eu(NO_3_)_3_ (18 mM) (red emission, D‐isomer) were initially prepared, and 30 µL of these mixtures were loaded into the 384‐well plate as per the design pattern (Figure 6B). Initially, the pH of the solution was lowered to ∼4.5 due to the presence of **Thr_D/L_**, which prevented the formation of the NCs. As a result, no pattern was visible under daylight at this stage (state 1). Subsequently, two different combinations of the chemical additives were prepared: (i) with ε‐caprolactone (250 µL), and (ii) with β‐butyrolactone (50 µL), keeping the triethylamine concentration (50 µL) the same in both cases. The chemical additive made of ε‐caprolactone was added to all wells of Tb(NO_3_)_3_ and **Thr_D/L_**, while the chemical additive containing β‐butyrolactone was added to all wells of Tb(NO_3_)_3_, **Thr_D_**, and Eu(NO_3_)_3_ (Figure S16). Introduction of the chemical additive suddenly raised the pH of the solution in all wells to ∼9.5, resulting in the spontaneous formation of green (**Tb‐Thr_D/L_‐G**), yellow (**Tb‐Thr_D_‐Y**), and red (**Tb‐Thr_D_‐R**) emissive NCs in their respective wells.

Accordingly, a complex pattern of green, yellow, and red luminescence was observed under the UV light (state 2), which was completely different from the actual encrypted information, “IISER”. Interestingly, β‐butyrolactone present in the wells of **Tb‐Thr_D_‐Y** and **Tb‐Thr_D_‐R** NCs underwent faster hydrolysis. This process caused the dissociation of **Tb‐Thr_D_‐Y** and **Tb‐Thr_D_‐R** NCs into their respective non‐luminescent precursors in ∼40 min. As a result, the yellow and red luminescence disappeared from the pattern. This subsequently led to the emergence of another incorrect pattern “U68A” due to green‐emissive **Tb‐Thr_D/L_‐G** NCs (state 3). Among the green‐emissive NCs, we have used D and L‐stereoisomers of **Thr,** and hence the correct pattern “IISER” can only be decrypted with the use of CPL detection, as the correct information was encrypted using the D‐stereoisomer (state 4) (Figure S16). The use of excited state chirality in encryption makes the material challenging to counterfeit. Finally, slow hydrolysis of the ε‐caprolactone present in **Tb‐Thr_D/L_‐G** NCs solution caused their dissociation into their corresponding non‐luminescent precursors within ∼120 min. This process led to the autonomous regeneration of the initial non‐emissive state (state 1), resulting in the complete self‐erasure of all the patterns. Our approach is unique in several aspects, including: (i) programmable erasure of encrypted information in a temporal manner after decryption, (ii) the requirement for four different keys for decryption (Figure 6C), which include a chemical additive, UV light irradiation, time, and CPL, (iii) multi‐level information storage using a single nano‐entity, thereby offering high security for the encrypted data, and (iv) the dynamic nature of the NCs, which allows for temporal control over the encryption and decryption of information in an autonomous fashion with high concealment efficiency. Our strategy also permits further improvement of the encrypted information by selecting different chemical additives and controlling their concentrations.

### 2.5 Anticounterfeiting Applications

**FIGURE 7:**
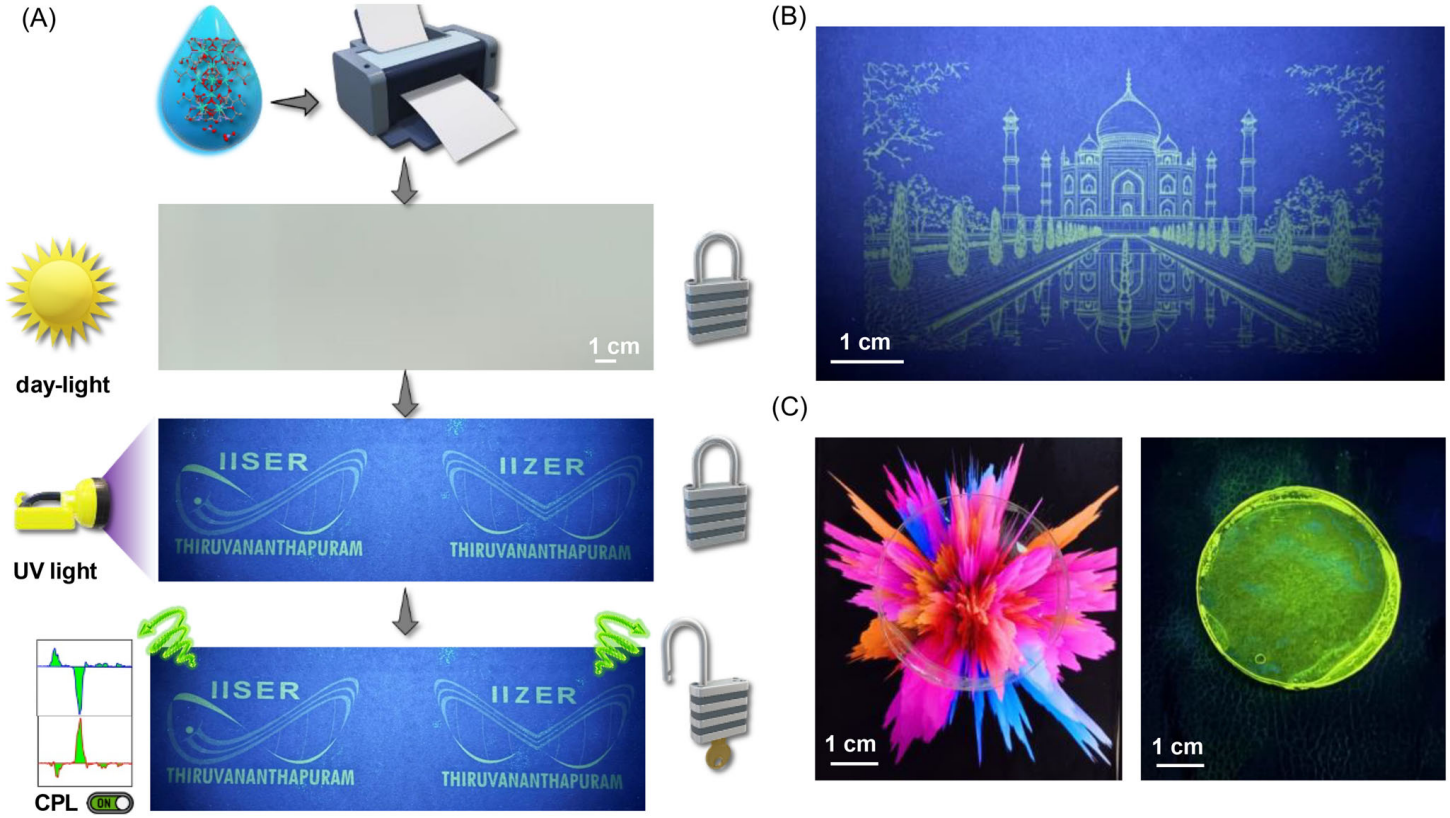
Tb‐Thr_D/L_‐G NCs as anticounterfeiting ink. (A) Schematic representation showing the printing using Tb‐Thr_D/L_‐G NCs in water as anticounterfeiting ink. Photographs of the printed images under UV light and with CPL detection (schematic). (B) Photograph of “Taj Mahal” printed using Tb‐Thr_D_‐G as the ink under UV light. (C) Photographs of Tb‐Thr_D_‐G‐PVA polymer composite under day (left) and UV light (right).

Anticounterfeit inks are among the most effective methods for preventing counterfeiting [5]. The encryption labels on banknotes, driver's licenses, passports, and other government‐issued legal documents are mainly based on different types of security inks [1, 39]. Encouraged by the high‐level data encryption enabled by NCs, we demonstrated their potential as a covert anticounterfeiting ink using a commercial inkjet printer (Figure 7A). For this purpose, we utilized green‐emissive **Tb‐Thr_D_‐G** NCs (20 mg/mL: Tb(NO_3_)_3_, 10 mg/mL: **Thr_D/L_**) in water, which was fully compatible with commercial Deskjet printers. Particle size is crucial for printing applications, as it must fit the printer's nozzle diameters; ideally, it should be <1 µm to increase the resolution of printed graphics or pattern [6]. The hydrodynamic radius of **Tb‐Thr_D_‐G** NCs was ∼ 25 nm in aqueous medium as evident from the dynamic light scattering analyses (DLS), making them an ideal ink for printing applications (Figure S17). Subsequently, the ink was loaded into a commercial HP DeskJet 2331 printer, and a set of authentic and counterfeit “IISER” logos were printed on paper (Figure 7A). As expected, the authentic and counterfeit “IISER” logos were invisible in daylight, but clearly visible under UV light, appearing as green emissive letters. As expected, the authentic and counterfeit “IISER” logos were invisible in daylight but clearly visible under UV light, appearing as a green emissive pattern. Furthermore, by exploring the CPL activity of the printed ink, two signals can be obtained for the two stereoisomers, thereby providing a higher level of protection (Figure S18). The use of **Tb‐Thr_D_‐G** NCs as ink also enabled the printing of highly complex patterns, making them a highly promising material as an anticounterfeiting ink (Figure 7B and Figure S19). Furthermore, **Tb‐Thr_D_‐G** NCs can be easily dispersed in commercially available polymers, resulting in nearly 100% transparent polymer composites, while maintaining the luminescent properties of the NCs. As a proof‐of‐concept, we used polyvinyl alcohol (**PVA**) as a polymer matrix to demonstrate the compatibility of the NCs with the polymer matrix. Interestingly, PVA showed excellent compatibility with **Tb‐Thr_D_‐G** NCs due to the possibility of multiple H‐bonding interactions between **PVA** and **Tb‐Thr_D_‐G** NCs. The **Tb‐Thr_D_‐G‐PVA** composite was fully transparent and flexible, with no traces of doping of the NCs (Figure 7C). In addition, the composite exhibited intense green emission of **Tb‐Thr_D_‐G** NCs under UV light irradiation (Figure 7C). Given the potential of **PVA** as a common matrix for various technological applications, we believe that the **Tb‐Thr_D_‐G‐PVA** composite would be a promising candidate for numerous real‐life applications.

## 3 Conclusions

In summary, we have reported a one‐pot, aqueous, and scalable synthesis of chiral lanthanide supramolecular material using terbium and threonine. They possess the essential features required for high‐level data encryption and serve as an anticounterfeiting agent within a single nanoentity. The key features of the aqueous solution of the NCs include: (i) nearly 100% transparency, (ii) tunable luminescence and CPL through controlled doping with Eu(III), (iii) a pH feedback loop‐controlled “on‐off” of luminescence and CPL, (iv) temporal control over encryption‐decryption, and (v) the ability to erase stored information autonomously. By harnessing the remarkable properties of these NCs, we have successfully demonstrated high‐level data encryption that operates entirely autonomously. Decrypting the information requires four different keys: UV light, a chemical additive, time, and CPL, which significantly enhances the security of the stored data. Furthermore, we have shown the potential of these NCs as a biocompatible anticounterfeiting ink for printing complex patterns on paper using a commercial inkjet printer, offering multiple levels of protection. The excellent compatibility of NCs with commercial polymer matrices is also promising for their technological applications. We firmly believe that our rational strategy for the scalable synthesis of terbium‐threonine NCs with remarkable optical and chiroptical properties in water using readily available materials will pave the way for the facile synthesis of new lanthanide‐based chiral emitters with promising applications.

## Author Contributions

**Jim Bachmann**: investigation, software, data curation. **Nidhin Ravi**: investigation, validation, data curation. **P. M. Ajay Sekhar**: investigation, validation, writing – original draft, formal analysis, data curation. **Nikos L. Doltsinis**: investigation, writing – review and editing, supervision. **Reji Varghese**: supervision, project administration, writing – review and editing, funding acquisition, conceptualization. **Kaloor S. Harikrishnan**: investigation, validation, data curation.

## Funding

Financial supports from the ANRF (ANRF/ARG/2025/007327/CS) and the IRTG (INT/FRG/IRTG/01/2024 (G)) gratefully acknowledged. P.M.A.S. and K.S.H. thank IISER Thiruvananthapuram for their PhD Fellowship.

## Conflicts of Interest

The authors declare no conflicts of interest.

## Supporting information




**Supporting File**: advs77336‐sup‐0001‐SuppMat.docx.

## Data Availability

The data that support the findings of this study are available from the corresponding author upon reasonable request.
