# Supplementary material for: Supramolecular Terbium‐Threonine Chiral Emitters with Tunable Circularly Polarized Luminescence as Anticounterfeiting‐Ink for Autonomous Data Encryption
Source: Adv Sci (Weinh). 2026 Aug 21;13(63):e77336. doi: 10.1002/advs.77336 (PMC13496157; doi:10.1002/advs.77336)
Supplement: Supplementary file 1 [file ADVS-13-e77336-s001.docx]

Supporting Information

Supramolecular Terbium-Threonine Chiral Emitters with Tunable Circularly Polarized Luminescence as Anticounterfeiting-Ink for Autonomous Data Encryption

P. M. Ajay Sekhar,^a^ Nidhin Ravi,^a^ Kaloor S. Harikrishnan,^a^ Jim Bachmann,^b^ Nikos L. Doltsinis,^2^* and Reji Varghese^a^*

|  | **TABLE OF CONTENTS** |
| --- | --- |
| **SI. No.** | **Contents** |
| 1 | Materials and methods |
| 2 | Synthesis methods |
| 3 | General characterization |
| 4 | Crystal data and structure refinement for **Tb-Thr_D_-G** NC |
| 5 | ORTEP diagram of **Tb-Thr_D_-G** NC |
| 6 | Hydrogen bonding interactions present in **Tb-Thr_D_-G** NC |
| 7 | CIE colour coordinate diagram of **Tb-Thr_D/L_-G** NCs luminescence |
| 8 | Optimized structures **Tb-Thr_D_-G** NC and its smaller model system |
| 9 | TDDFT spectra of **Tb-Thr_D_-G** NC and model system |
| 10 | TDDFT data for model system |
| 11 | TDDFT Dominant orbitals for model system |
| 12 | TDDFT Dominant orbitals for **Tb-Thr_D_-G** NC |
| 13 | TDDFT data for **Tb-Thr_D_-G** NC |
| 14 | Absorption and CD spectra of **Tb-Thr_D/L_-Y** and **Tb-Thr_D/_L-R** NCs |
| 15 | Comparison of PXRD patterns of the NCs |
| 16 | Time resolved luminescence spectra of **Tb-Thr_D_-Y** and **Tb-Thr_D_-R** NCs |
| 17 | Comparison of optical properties of the NCs in the solid and solution states |
| 18 | Chiroptical properties of the NCs in the solid state |
| 19 | Plot of average Tb-Tb distance of all Tb atom during the xTB simulation |
| 20 | Single crystals of threonine obtained at pH 5.5 and 7.5 |
| 21 | CPL spectra of **Tb-Thr_L_-G** NCs at different pH |
| 22 | Comparison of the XPS spectra of threonine and **Tb-Thr_D_-G** NCs |
| 23 | Design pattern for the multi-level data encryption |
| 24 | DLS size distribution of **Tb-Thr_D_-G** NCs in water |
| 25 | Images of the print using **Tb-Thr_D/L-_G** as anti-counterfeiting ink |
| 26 | Different complex patterns printed using **Tb-Thr_D_-G** NCs |
| 27 | Details of cartridge refill steps |
| 28 | References |

**Materials and Methods**

**Material****s**

Tb(NO_3_)_3_ .5H_2_O (99.9 %, SRL)_,_ Eu(NO_3_)_3_.5H_2_O (99.9 %, SRL)_,_ caprolactone (99.93 %, BLD chemicals), β-butyrolactone (98 %, Sigma-Aldrich), D-(+)-glucono-1,5-lactone(98 %, TCI), triethylamine(99 %, Spectrochem), D/L-threonine (98 %, BLD Chemicals), poly(vinyl alcohol) (Mw = ~150,000, Sigma-Aldrich) were used as received without any further purification. Deionized water was utilized throughout the whole experimental process.

**Synthesis methods**

**Tb-Thr_D/L_-G NCs**: **Tb-Thr_D/L_-G** NCs were synthesized by the mixing of **Thr_D/L_** (5.47 mg, 0.045 mmol, 2 eq.) and Tb(NO_3_)_3_ (10 mg, 0.023 mmol, 1 eq.) at basic pH (~9.5) in deionized water at 25 °C. The pH of the solution was made to ~9.5 by the addition of triethylamine and the NCs were formed spontaneously. The single crystals were grown over four weeks via the solvent diffusion method by the slow diffusion of acetone into the solution of NCs in water (pH ~9.5) at 25 °C.

**Tb-Thr_D/L_-Y NCs**: **Tb-Thr_D/L_-Y** NCs were synthesized by the mixing of **Thr_D/L_** (5.47 mg, 0.045 mmol, 2 eq.), Tb(NO_3_)_3_ (9.487 mg, 0.0218 mmol, 0.95 eq.) and Eu(NO_3_)_3_ (0.5125 mg, 0.0011 mmol, 0.05 eq.) in deionized water (pH ~9.5) at 25 °C.

**Tb-Thr_D/L_-R NCs**: **Tb-Thr_D/L_-R** NCs were synthesized by the mixing of **Thr_D/L_** (5.47 mg, 0.045 mmol, 2 eq.), Tb(NO_3_)_3_ (8.975 mg, 0.02 mmol, 0.90 eq.) and Eu(NO_3_)_3_ (1.025 mg, 0.002 mmol, 0.1 eq.) in deionized water (pH ~9.5) at 25 °C.

**Tb-Thr_D/L_-G-PVA composite**: Stock PVA solution was prepared by using 1.5 g of the polymer in 30 mL of deionized water, followed by heating at 80 °C for 1 h under stirring conditions. Subsequently, **Tb-Thr_D/L_-G-PVA** polymer composite was synthesized by adding 1 mL of **Tb-Thr_D/L_-G** NCs (20 mM) into 3 mL of PVA stock solution, followed by the sonication and heating at 80 °C for 30 minutes to get a homogeneous solution of **Tb-Thr_D/L_-G-PVA** composite. This was then drop-casted on a clean glass substrate for further experiments.

**General characterization**

Single crystal X-ray diffraction data was recorded using Bruker D8 Quest-Microfocus SC-XRD with graphite-monochromatized Mo Kα radiation (λ = 0.71073 Å). Bruker Apex III and V software Suite were used for data reduction, while the structure solution was done with SHELXT-2014. NMR measurements were done on 500 MHz Bruker Avance III NMR spectrometer. pH measurements were carried out using Horiba LAQUA F-74BW pH meter. All pH measurements were carried out at 25 °C. Absorption/transmittance and emission measurements were recorded on a Shimadzu UV-3600 UV-Vis spectrophotometer, and Horiba Jobin Yvon-Fluorolog spectrofluorometer, respectively using a 10 mm pathlength quartz cuvette. CD analyses were carried out on a JASCO J-815 CD spectropolarimeter having Peltier controlled thermostatic cell holder. CPL spectra were measured on a JASCO CPL-300 circularly polarized luminescence spectrophotometer. Both CD and CPL measurements were done in a quartz cuvette of 10 mm path length. Edinburgh FLS-1000 fluorescence spectrophotometer with a xenon arc lamp (Xe 900) and a microsecond flash-lamp (µF 1000) was used to determine the absolute photoluminescence quantum yields using integrating sphere. The photoluminescence life time measurements were done on a Horiba Jobin Yvon-Fluorolog spectrofluorometer. TEM analyses were conducted on FEI Tecnai (300 kV) electron microscope. For the TEM analyses, 5 μL of the sample in water was deposited on a 400-mesh carbon-coated copper grid (Ted Pella, Inc.), and then allowed to adsorb onto the grid for 2 minutes. The excess sample was removed by softly touching the sample droplet with a piece of filter paper and then the grid was allowed to dry under air. TDDFT calculations were performed using the CP2K software,^1,2^ offering the required computational speed combined with a highly flexible Gaussian and Plane Wave (GPW) basis set. For H, O, N, C and Tb DZVP basis sets were utilized together with a plane wave cutoff of 600 Ry and relativistic, separable Goedecker-Teter-Hutter Gaussian pseudopotentials.^3^ The generalized gradient exchange-correlation functional PBE^4^ was employed together with the Grimme-D3^5^ dispersion correction. Due to the high density of electronic states of Tb, Fermi-Dirac smearing with a temperature of 300K was enabled. A total of 5000 excited states were calculated for the cluster (60 states for the smaller model system).

| CCDC number | 2538231 |  |  |  |
| --- | --- | --- | --- | --- |
| Empirical formula | C_80_H_238_N_22_O_116_Tb_14_ | |  |  |
| Formula weight | 5589.79 |  |  |  |
| Temperature | 100(2) K |  |  |  |
| Wavelength | 0.71073 Å |  |  |  |
| Crystal system | Orthorhombic |  |  |  |
| Space group | P2**_1_**2**_1_**2 |  |  |  |
| Unit cell dimensions | a = 30.7002(18) Å | α = 90° |  |  |
|  | b = 30.8097(16) Å | β = 90° |  |  |
|  | c = 19.1051(9) Å | γ = 90° |  |  |
| Volume | 18070.8(17) Å^3^ |  |  |  |
| Z | 4 |  |  |  |
| Density (calculated) | 2.055 Mg/m^3^ |  |  |  |
| Absorption coefficient | 5.509 mm^-1^ |  |  |  |
| F(000) | 10840 |  |  |  |
| Crystal size | 0.150 x 0.140 x 0.100 mm^3^ | |  |  |
| Theta range for data collection | 1.823 to 28.313°. |  |  |  |
| Index ranges | -40<=h<=40, -41<=k<=41, -25<=l<=25 | | |  |
| Reflections collected | 1059950 |  |  |  |
| Independent reflections | 44904 [R(int) = 0.0627] | |  |  |
| Completeness to theta = 25.242° | 99.9 % |  |  |  |
| Absorption correction | Semi-empirical from equivalents | |  |  |
| Max. and min. transmission | 0.54 and 0.39 |  |  |  |
| Refinement method | Full-matrix least-squares on F^2^ | |  |  |
| Data / restraints / parameters | 44904 / 778 / 2350 |  |  |  |
| Goodness-of-fit on F^2^ | 1.206 |  |  |  |
| Final R indices [I>2sigma(I)] | R1 = 0.0489, wR2 = 0.1104 | |  |  |
| R indices (all data) | R1 = 0.0529, wR2 = 0.1137 | |  |  |
| Absolute structure parameter | 0.023(2) |  |  |  |
| Extinction coefficient | n/a |  |  |  |
| Largest diff. peak and hole | 3.027 and -2.280 e. Å -3 | |  |  |

**Table S1.** Crystal data and structure refinement for **Tb-Thr_D_-G** NC.

**Figure S1**. ORTEP diagram of **Tb-Thr_D_-G** NC.

____________________________________________________________________________

**D-H...A	d(D-H)	d(H...A)	d(D...A)	<(DHA)**

____________________________________________________________________________

C(1)-H(1B)...O(1A)	0.98	2.59	3.33(4)	132.1

C(1)-H(1B)...O(2A)	0.98	2.54	3.52(4)	177.9

C(1)-H(1B)...N(1A)	0.98	2.56	3.45(3)	151.2

C(3)-H(3)...O(53^c)#1	1.00	2.51	3.23(3)	128.3

C(9)-H(9B)...O(93)	0.98	2.51	3.34(4)	141.9

C(11)-H(11)...O(47)#1	1.00	2.56	3.31(2)	131.8

C(13)-H(13B)...O(88)	0.98	2.64	3.53(3)	149.9

C(15)-H(15)...O(40)#2	1.00	2.40	3.18(2)	134.3

C(23)-H(23)...O(49)#3	1.00	2.51	3.35(2)	141.4

C(26)-H(26)...O(44)#1	1.00	2.62	3.614(19)	171.0

C(28)-H(28A)...O(2A)#4	0.98	2.56	3.34(4)	136.4

C(28)-H(28B)...O(90)	0.98	2.57	3.45(4)	149.7

C(33)-H(33C)...O(26)	0.98	2.64	3.20(3)	116.7

C(33)-H(33C)...O(37^a)	0.98	2.56	3.51(4)	162.1

C(41)-H(41C)...O(32)	0.98	2.49	3.25(2)	133.7

C(51)-H(51^a)...O(43)	1.00	2.56	3.17(2)	119.3

C(53)-H(53A)...O(32)	0.98	2.51	3.29(2)	136.9

C(53)-H(53A)...O(105)	0.98	2.48	3.36(6)	148.8

C(53)-H(53B)...O(41)	0.98	2.64	3.19(2)	115.9

C(59)-H(59)...O(20)#5	1.00	2.61	3.21(2)	118.3

C(61)-H(61B)...O(3A)#5	0.98	2.42	3.39(5)	168.8

C(61)-H(61C)...O(107)	0.98	2.57	3.28(4)	128.9

C(63)-H(63)...O(8)#5	1.00	2.46	3.40(2)	156.3

C(65)-H(65C)...O(29)	0.98	2.59	3.33(3)	131.9

C(65)-H(65C)...O(110)	0.98	2.62	3.46(6)	143.2

C(67)-H(67)...O(46)	1.00	2.60	3.19(2)	117.7

C(69)-H(69B)...O(91)	0.98	2.61	3.57(4)	167.6

C(70)-H(70)...O(116)	1.00	2.47	3.39(5)	152.4

C(74)-H(74)...O(105)	1.00	2.61	3.49(6)	147.7

C(77)-H(77A)...O(92)#3	0.98	2.54	3.44(3)	153.2

O(1)-H(1)...O(92)	0.84	2.03	2.64(2)	129.7

O(4)-H(4)...O(25)	0.84	1.83	2.637(19)	160.5

O(7)-H(7A)...O(89)	0.84	1.92	2.69(2)	152.7

O(10)-H(10C)...O(34)	0.84	2.27	2.80(3)	121.1

O(10)-H(10C)...O(113)	0.84	2.13	2.83(5)	141.2

O(13)-H(13)...O(102)	0.84	1.95	2.69(2)	146.6

O(16)-H(16)...O(101)	0.84	1.93	2.75(6)	167.3

O(19)-H(19A)...O(87)	0.84	1.92	2.650(19)	144.6

O(22)-H(22A)...O(23)	0.84	2.48	3.010(18)	122.2

O(22)-H(22A)...O(82)	0.84	2.07	2.570(17)	117.9

O(25)-H(25)...O(31)#6	0.84	1.98	2.72(2)	146.2

O(28)-H(28)...O(22)	0.84	1.85	2.646(19)	158.6

O(31)-H(31A)...O(16)	0.84	2.15	2.86(2)	141.7

O(34)-H(34A)...O(28)#2	0.84	2.03	2.70(2)	136.5

C(49^a)-H(49A^a)...O(38)	0.98	2.06	2.69(4)	120.7

C(49^a)-H(49B^a)...O(102)#2	0.98	1.74	2.71(5)	174.0

O(37^a)-H(37^a)...O(26)	0.84	2.50	3.25(3)	148.5

O(37^a)-H(37^a)...O(100)#6	0.84	2.45	3.03(5)	127.5

C(49'^b)-H(49D^b)...O(38)	0.98	2.25	2.96(6)	128.5

C(49'^b)-H(49E^b)...O(13)#2	0.98	2.61	3.53(7)	157.3

C(49'^b)-H(49F^b)...O(102)#2	0.98	1.94	2.51(7)	114.2

O(37'^b)-H(37'^b)...N(13)	0.84	2.18	2.63(4)	113.5

O(40)-H(40)...O(89)#7	0.84	2.08	2.89(2)	161.7

O(43)-H(43A)...O(35)	0.84	2.14	2.677(16)	121.9

O(46)-H(46A)...O(26)	0.84	2.07	2.653(19)	126.3

O(49)-H(49)...O(50)	0.84	2.17	2.707(18)	122.0

O(52)-H(52)...O(29)	0.84	1.96	2.604(17)	132.3

O(55)-H(55A)...O(32)	0.84	2.24	2.728(16)	117.6

O(58)-H(58A)...O(35)	0.84	2.15	2.935(19)	154.8

O(63)-H(63A)...O(82)	1.00	1.82	2.778(16)	159.8

O(64)-H(64)...O(84)	1.00	1.84	2.815(16)	164.0

O(65)-H(65)...O(86)	1.00	1.86	2.819(16)	160.0

O(66)-H(66A)...O(80)	1.00	1.85	2.819(16)	160.8

O(67)-H(67A)...O(24)	1.00	1.84	2.788(15)	156.4

O(68)-H(68)...O(6)	1.00	1.85	2.791(15)	156.4

O(69)-H(69)...O(11)	1.00	2.63	3.507(17)	146.8

O(69)-H(69)...O(12)	1.00	1.77	2.710(15)	155.7

O(70)-H(70A)...O(18)	1.00	1.80	2.749(14)	156.9

O(71)-H(71A)...O(42)	1.00	1.78	2.755(14)	163.2

O(72)-H(72)...O(60)	1.00	1.78	2.745(13)	162.2

O(73)-H(73)...O(39)	1.00	1.77	2.737(14)	161.2

O(74)-H(74A)...O(51)	1.00	1.81	2.769(13)	160.7

O(75)-H(75A)...O(32)	1.00	2.64	3.459(16)	138.8

O(75)-H(75A)...O(33)	1.00	1.78	2.753(15)	164.4

O(76)-H(76)...O(36)	1.00	1.81	2.782(14)	162.8

O(77)-H(77)...O(27)	1.00	1.85	2.817(16)	161.8

O(78)-H(78A)...O(30)	1.00	1.83	2.801(16)	161.7

N(1)-H(1D)...O(53'^d)#1	0.91	2.30	2.96(3)	129.5

N(1)-H(1D)...O(4A^e)	0.91	2.44	3.24(3)	146.5

N(1)-H(1E)...O(8)	0.91	2.36	2.91(2)	119.2

N(2)-H(2A)...O(8)	0.91	2.32	2.962(18)	127.0

N(2)-H(2B)...O(4)	0.91	2.42	2.875(19)	111.4

N(3)-H(3B)...O(93)	0.91	2.40	3.27(4)	160.9

N(4)-H(4A)...O(20)	0.91	2.61	3.17(2)	120.2

N(5)-H(5D)...O(4A^e)	0.91	2.24	3.10(3)	157.7

N(5)-H(5D)...O(4B^f)	0.91	2.17	2.96(3)	143.5

N(5)-H(5E)...O(2)	0.91	2.40	3.04(2)	126.6

N(6)-H(6A)...O(90)	0.91	2.32	3.22(2)	171.3

N(6)-H(6B)...O(16)	0.91	2.43	2.87(2)	110.3

N(7)-H(7D)...O(5A^e)	0.91	2.22	3.10(3)	161.1

N(7)-H(7D)...O(4B^f)	0.91	2.64	3.49(4)	155.1

N(7)-H(7E)...O(90)	0.91	2.14	3.04(2)	170.0

N(8)-H(8A)...O(2)	0.91	2.40	3.034(17)	126.9

N(9)-H(9D)...O(50)	0.91	2.22	3.06(2)	154.0

N(9)-H(9D)...O(81)	0.91	2.61	3.320(19)	135.5

N(9)-H(9E)...O(5)	0.91	2.41	3.049(18)	127.7

N(9)-H(9E)...O(25)	0.91	2.36	2.82(2)	111.1

N(10)-H(10A)...O(41)	0.91	2.18	3.021(18)	153.3

N(10)-H(10B)...O(22)	0.91	2.63	3.479(19)	156.0

N(10)-H(10B)...O(23)	0.91	2.47	3.093(17)	126.4

N(10)-H(10B)...O(28)	0.91	2.37	2.84(2)	112.0

N(11)-H(11A)...O(59)	0.91	2.10	2.947(19)	154.5

N(11)-H(11B)...O(17)	0.91	2.58	3.171(19)	123.0

N(11)-H(11B)...O(31)	0.91	2.35	2.815(19)	111.8

N(12)-H(12A)...O(38)	0.91	2.19	3.06(2)	159.0

N(12)-H(12B)...O(11)	0.91	2.39	3.025(19)	126.4

N(12)-H(12B)...O(34)	0.91	2.33	2.79(2)	110.9

N(13)-H(13D)...O(47)	0.91	2.54	3.18(2)	128.0

N(13)-H(13D)...O(95)	0.91	2.62	3.51(4)	166.0

N(13)-H(13E)...O(26)	0.91	2.56	3.46(2)	170.1

N(14)-H(14A)...O(56)	0.91	2.29	2.937(19)	127.4

N(14)-H(14A)...O(91)	0.91	2.39	3.17(3)	143.1

N(14)-H(14B)...O(32)	0.91	2.52	3.42(2)	171.1

N(15)-H(15A)...O(20)#5	0.91	2.58	3.201(19)	126.3

N(15)-H(15A)...O(6A^e)#5	0.91	2.62	3.43(2)	147.7

N(15)-H(15A)...O(5B^f)#5	0.91	2.57	3.30(3)	137.0

N(15)-H(15B)...O(95)	0.91	2.50	3.39(4)	165.6

N(16)-H(16A)...O(53'^d)	0.91	2.15	2.75(4)	122.7

N(16)-H(16A)...O(6B^f)#5	0.91	2.57	3.41(3)	153.1

N(16)-H(16B)...O(3A)#5	0.91	2.43	3.33(3)	168.5

N(17)-H(17D)...O(53^c)	0.91	2.37	3.01(3)	127.5

N(17)-H(17D)...O(3A)#5	0.91	2.29	3.11(3)	148.7

N(17)-H(17E)...O(29)	0.91	2.61	3.51(2)	169.7

N(18)-H(18A)...O(5A^e)#5	0.91	2.64	3.48(3)	152.8

N(18)-H(18A)...O(4B^f)#5	0.91	2.28	3.13(4)	154.6

N(18)-H(18B)...O(91)	0.91	2.30	3.21(3)	174.9

N(19)-H(19D)...O(5A^e)#5	0.91	2.40	3.18(3)	142.6

N(19)-H(19D)...O(5B^f)#5	0.91	2.40	3.23(3)	152.0

N(19)-H(19E)...O(44)	0.91	2.41	2.96(2)	119.0

N(20)-H(20A)...O(44)	0.91	2.31	2.970(18)	129.3

N(20)-H(20B)...O(35)	0.91	2.41	3.290(18)	163.8

O(79)-H(79A)...O(38)	0.77(2)	2.07(3)	2.783(18)	153(6)

O(79)-H(79B)...O(11)	0.82(3)	2.10(3)	2.740(17)	135(5)

O(80)-H(80A)...O(12)	0.87(2)	2.54(5)	3.000(18)	114(4)

O(80)-H(80A)...O(89)	0.87(2)	2.21(3)	3.04(2)	159(5)

O(80)-H(80B)...O(4)	0.86(2)	1.78(3)	2.610(17)	163(5)

O(81)-H(81A)...O(50)	0.90(2)	2.12(3)	2.798(16)	132(4)

O(81)-H(81B)...O(5)	0.86(3)	2.07(3)	2.709(17)	130(4)

O(81)-H(81B)...N(9)	0.86(3)	2.56(5)	3.320(19)	147(4)

O(82)-H(82A)...O(6)	0.947(14)	2.10(2)	2.942(18)	147.4(17)

O(82)-H(82B)...O(92)	0.887(14)	2.66(5)	3.27(2)	127(5)

O(83)-H(83A)...O(23)	0.853(14)	2.19(16)	2.740(18)	122(15)

O(83)-H(83B)...O(41)	0.854(14)	1.96(5)	2.792(17)	163(16)

O(84)-H(84A)...O(102)	0.86(3)	2.11(4)	2.86(2)	144(6)

O(84)-H(84B)...O(16)	0.85(3)	1.90(5)	2.686(19)	155(8)

O(85)-H(85A)...O(59)	0.90(2)	2.13(3)	2.755(16)	126(3)

O(85)-H(85B)...O(17)	0.90(3)	2.08(3)	2.672(18)	123(4)

O(85)-H(85B)...N(11)	0.90(3)	2.62(4)	3.429(18)	151(4)

O(86)-H(86A)...O(10)	0.84(3)	1.90(3)	2.704(19)	160(6)

O(86)-H(86B)...O(85)	0.94(3)	2.21(2)	2.783(16)	119(2)

O(86)-H(86B)...O(87)	0.94(3)	2.41(3)	3.074(19)	128(3)

O(87)-H(87A)...O(113)	0.90	1.95	2.69(5)	138.7

O(87)-H(87B)...O(17)	0.90	2.55	3.29(2)	139.5

O(87)-H(87B)...O(49)#3	0.90	2.20	2.81(2)	124.2

O(88)-H(88A)...O(93)	0.97	1.79	2.75(4)	169.1

O(88)-H(88B)...O(20)	0.91	1.99	2.75(3)	141.0

O(90)-H(90A)...O(1A)#4	0.88	2.24	2.88(3)	129.8

O(90)-H(90B)...O(107)#3	0.87	2.06	2.86(4)	152.9

O(94)-H(94A)...O(44)	0.90	2.07	2.79(3)	136.0

O(94)-H(94B)...O(108)#3	0.90	2.23	2.98(4)	139.9

O(97)-H(97A)...O(88)	0.90	1.84	2.71(4)	160.0

O(97)-H(97B)...O(94)#1	0.90	2.13	2.73(4)	122.7

O(98)-H(98A)...O(8)	0.90	2.21	2.85(3)	127.6

O(98)-H(98B)...O(107)#1	0.90	2.25	2.94(4)	132.8

____________________________________________________________________________

**Table S2.** Hydrogen bonding interactions present in **Tb-Thr_D_-G** NC [Å and °].

**Figure S2**. The CIE colour coordinate diagram of **Tb-Thr_D_-G** (blue) and **Tb-Thr_L_-G** (red) NC luminescence.

**Figure S3**. Optimized structures **Tb-Thr_D_-G** NC (left) and its smaller model system (right).

**Figure S4**. TDDFT spectra of **Tb-Thr_D_-G** NC (left) and the corresponding smaller model system (right). A gaussian broadening with a FWHM of 35 nm has been applied. The red dots are locations of the individual transitions, where a lot of dark states are observed in the NC. The blue transitions contribute the most to the absorption bands. Two bands are predicted for the absorption spectrum of **Tb-Thr_D_-G** NCs centred at 250 nm and 300 nm.

**Table S3.** TDDFT data for the dominant transitions in the model complex shown in Fig. S3. To assess the orbitals involved in these bands the most dominant orbitals taking part in these transitions are plotted in Figure S5.

**Figure S5**. Dominant orbitals in the small model system contributing to the 300 nm (a) and 250 nm (b) absorption bands. The excitations promote electrons from the ligands to **Tb** d-orbitals.

**Figure S6**. Dominant orbital transitions contributing to the absorption band at 300 nm. The transitions go from ligand orbitals to several **Tb** d-orbitals, which shows that multiple **Tb** atoms are involved in the absorption. Here it can clearly be seen that the transition goes from the ligand to **Tb** d-orbitals and ligand orbitals.

**Table S4**. TDDFT data for the most dominant transitions contributing to the absorption bands at 300 nm and 250 nm for **Tb-Thr_D_-G** NCs.

**Figure S7**. Absorption spectra of (A) **Tb-Thr_D/L_-Y** and (B) **Tb-Thr_D/L_-R** NCs in water. CD spectra of (C) **Tb-Thr_D/L_-Y** and (D) **Tb-Thr_D/L_-R** NCs in water.

**Figure S8**. Comparison of PXRD patterns of **Tb-Thr_D_-G**, **Tb-Thr_D_-Y**, **Tb-Thr_D_-R** and simulated PXRD from the single crystal X-ray diffraction of **Tb-Thr_D_-G** NCs.

**Figure S9**. Time resolved luminescence spectra of (A) **Tb-Thr_D_-Y** and (B) **Tb-Thr_D_-R** NCs.

**Figure S10**. Comparison of optical properties of the NCs in solid and solution states. (A-C) Absorption spectra of the NCs in solid phase and the respective insets show the corresponding spectra in the solution state. (D-F) Luminescence spectra of the NCs (λ_exc_ = 255 nm) in solid state and respective insets show the corresponding spectra in the solution state.

**Figure S11**. Chiroptical properties of the NCs in the solid state. (A-C) CD and (D-F) CPL (λ_exc_ = 255 nm) spectra of the NCs in the solid state.

**Figure S12**. Plot of average Tb-Tb distance of all Tb atom during the xTB simulation.

**Figure S13**: Single crystals of threonine obtained at pH (A) 5.5 and (B) 7.5.

**Figure S14**. CPL spectra of **Tb-Thr_L_-G** NCs in water (λ_exc_ = 255 nm) at different pH (~5.5, ~7.5, and ~9.5).

**Figure S15**. Comparison of the XPS spectra of threonine (A and B) and **Tb-Thr_D_-G** NCs (C and D).

**Figure S16**. Design for the multi-level data encryption patterns of states 2, 3, and 4.

**Figure S17**. DLS size distribution of **Tb-Thr_D_-G** NCs in water.

**Figure S18**. Images of the print using **Tb-Thr_D_-G** and **Tb-Thr_L_-G** as anti-counterfeiting inks.

**Figure S19**. Different complex patterns printed using **Tb-Thr_D_-G** NCs as anti-counterfeiting ink under UV light irradiation.

**Figure S20**. **Cartridge refill steps.** (a) Disassembly of the black cartridge: The process of ink refilling starts by opening the cartridge’s upper casing to expose the internal black ink compartment. (b) Ink storage sponge removal: The black ink storage sponges were carefully removed from the cartridge. (c) Cleaning of the cartridge and sponge: The empty plastic cartridge and the removed sponge were cleaned with water as well as ethanol until no more ink was left out. It was noted that little black color still remain on the sponges, however, it won't affect the quality of the new ink. (d) Refill of the ink with luminescent property: **Tb-Thr_D/L_-G** NCs solution was injected into their corresponding sponges until they were saturated (approximately 3 mL). (e) Reassembly and closure of the cartridge : The refilled sponges were placed back into their correct positions within the cartridge, and the unit was completely sealed. Now the ink-loaded printer is ready for printing.

**References**

(1) Kühne, T. D.; Iannuzzi, M.; Del Ben, M.; Rybkin, V. V.; Seewald, P.; Stein, F.; Laino, T.; Khaliullin, R. Z.; Schütt, O.; Schiffmann, F.CP2K: An electronic structure and molecular dynamics software package - Quickstep: Efficient and accurate electronic structure calculations. *J. Chem. Phys.* **2020**, *152*, 194103.
(2) Hutter, J.; Iannuzzi, M.; Schiffmann, F.; VandeVondele, J. Cp2k: Atomistic Simulations of Condensed Matter Systems. *WIREs Comput. Mol. Sci.* **2014**, *4*, 15–25.
(3) Hartwigsen, C.; Goedecker, S.; Hutter, J. Relativistic separable dual-space Gaussian pseudopotentials from H to Rn. *Phys. Rev. B* **1998**, *58*, 3641–3662.
(4) Perdew, J. P.; Burke, K.; Ernzerhof, M. Generalized Gradient Approximation Made Simple. *Phys. Rev. Lett.* **1996**, *77*, 3865–3868.
(5) Grimme, S.; Antony, J.; Ehrlich, S.; Krieg, H. A Consistent and Accurate ab initio Parametrization of Density Functional Dispersion Correction (DFT-D) for the 94 Elements H-Pu. *J. Chem. Phys.* **2010**, *132*, 154104.
